# Identification of Endotypes of Hospitalized COVID-19 Patients

**DOI:** 10.3389/fmed.2021.770343

**Published:** 2021-11-11

**Authors:** Benjamin L. Ranard, Murad Megjhani, Kalijah Terilli, Kevin Doyle, Jan Claassen, Michael R. Pinsky, Gilles Clermont, Yoram Vodovotz, Shadnaz Asgari, Soojin Park

**Affiliations:** ^1^Division of Pulmonary, Allergy, and Critical Care Medicine, Department of Medicine, NewYork-Presbyterian Hospital/Columbia University Irving Medical Center, New York, NY, United States; ^2^Program for Hospital and Intensive Care Informatics, Department of Neurology, Columbia University Irving Medical Center, New York, NY, United States; ^3^Department of Neurology, NewYork-Presbyterian Hospital/Columbia University Irving Medical Center, New York, NY, United States; ^4^Department of Critical Care Medicine, University of Pittsburgh, Pittsburgh, PA, United States; ^5^Department of Surgery, University of Pittsburgh, Pittsburgh, PA, United States; ^6^Department of Biomedical Engineering, California State University Long Beach, Long Beach, CA, United States

**Keywords:** COVID-19, cluster analysis, endotype, phenotype, machine learning, treatment, survival

## Abstract

**Background:** Characterization of coronavirus disease 2019 (COVID-19) endotypes may help explain variable clinical presentations and response to treatments. While risk factors for COVID-19 have been described, COVID-19 endotypes have not been elucidated.

**Objectives:** We sought to identify and describe COVID-19 endotypes of hospitalized patients.

**Methods:** Consensus clustering (using the ensemble method) of patient age and laboratory values during admission identified endotypes. We analyzed data from 528 patients with COVID-19 who were admitted to telemetry capable beds at Columbia University Irving Medical Center and discharged between March 12 to July 15, 2020.

**Results:** Four unique endotypes were identified and described by laboratory values, demographics, outcomes, and treatments. Endotypes 1 and 2 were comprised of low numbers of intubated patients (1 and 6%) and exhibited low mortality (1 and 6%), whereas endotypes 3 and 4 included high numbers of intubated patients (72 and 85%) with elevated mortality (21 and 43%). Endotypes 2 and 4 had the most comorbidities. Endotype 1 patients had low levels of inflammatory markers (ferritin, IL-6, CRP, LDH), low infectious markers (WBC, procalcitonin), and low degree of coagulopathy (PTT, PT), while endotype 4 had higher levels of those markers.

**Conclusions:** Four unique endotypes of hospitalized patients with COVID-19 were identified, which segregated patients based on inflammatory markers, infectious markers, evidence of end-organ dysfunction, comorbidities, and outcomes. High comorbidities did not associate with poor outcome endotypes. Further work is needed to validate these endotypes in other cohorts and to study endotype differences to treatment responses.

## Introduction

Coronavirus disease 2019 (COVID-19), caused by severe acute respiratory syndrome coronavirus 2 (SARS-COV-2), has demonstrated a wide variety of clinical courses, including asymptomatic carriers (1), mild disease (2), brief hospitalizations (2), prolonged ICU courses (3, 4), and COVID-19 “long-haulers” with prolonged symptoms (5). The spectrum of disease seems broader than the spectrum caused by other respiratory viruses, such as non-SARS-COV-2 coronaviruses. The international scientific community is currently endeavoring to understand the biological constructs that influence the course of disease after COVID-19 infection. Improved understanding of the biological underpinnings of different COVID-19 courses could improve diagnosis, triage, management, and prognosis for patients.

Several patient characteristics are associated with more severe COVID-19 disease or worse outcomes, including older age (3, 6), male sex (3, 6), obesity (7), diabetes mellitus (DM) (8), cardiovascular disease (3, 6), chronic obstructive pulmonary disease (COPD) (9), and chronic kidney disease (CKD) (10). Knowledge of baseline characteristics (including demographics and/or initial laboratory values) can predict hospitalization and mortality (11). There may be a subset of patients with a hyperinflammatory response who are at increased risk of mortality (12, 13).

Understanding endotypes of disease can shed light on biological underpinnings of disease and identify those who are most susceptible. Endotypes are subtypes of a clinical condition which possess distinct functional or pathobiological mechanisms (with an implicit variable likelihood of response to therapies across endotypes). It is envisaged that patients with a specific endotype present themselves within phenotypic clusters of disease, and because of the mechanistic differentiation, show response to specific therapies. Endotypes consist of subsets of the disease itself, rather than biological constructs which may or may not progress to disease (14). This approach has been used to describe subgroups in asthma (15), sepsis (16–19), trauma (20), and acute respiratory distress syndrome (ARDS) (21).

In clinical practice, baseline comorbidities and/or initial lab values do not explain the full range of COVID-19 presentations that are seen. We hypothesize that COVID-19 endotypes identified based on observable characteristics of the entire hospitalization (age and a representation of laboratory values) will reveal unexpected clinical courses and outcomes that defy prediction using classic risk factors. This approach is in contrast to some initial reports of clustering COVID-19 patients including using initial laboratory values and clinical variables collected in the first 24 (22) and 72 h (23); clustering patients by demographics, comorbidity, and maximum laboratory value (24) and using principal component analysis (PCA) and k-means of 18 initial laboratory values resulting in six values used in final analysis (25). Additionally, clusters have been created from initial ICU clinical data for patients with COVID-19 ARDS (26) and from ICU patients using demographics, initial ICU labs, and other clinical variables (27). Finally, there have been descriptions of a hyperinflammatory phenotype identified by initial admission labs (28) or serial labs using cluster analysis of three laboratory values (29).

In this study, we sought to uncover endotypes of the hospitalized COVID-19 patient population using a robust clustering method (consensus clustering of ensemble classification) on patient age and laboratory values over the course of hospital admission. These endotypes were examined for insights into comorbidities, expected clinical courses, and outcomes including intubation, length of stay (LOS), and mortality.

## Methods

### Participants

Adults (18 years-old or older) admitted consecutively to a telemetry capable bed at NewYork-Presbyterian Hospital/Columbia University Irving Medical Center were included in the study if they had a positive SARS-COV-2 nasopharyngeal PCR test during their inpatient admission and were discharged between March 12, 2020 to July 15, 2020. Patients with multiple admissions with a positive SARS-COV-2 nasopharyngeal PCR test only had data included from the first admission. If a patient had a positive SARS-COV-2 test (any type) more than 21 days before the admission, the patient was excluded. Patients were identified prospectively for inclusion in the study cohort but had their laboratory information, outcomes, and past medical history retrospectively collected. The collection of clinical data was done before clustering, so the investigators were blinded to endotype at the time of data collection. This study was approved by the Columbia University Institutional Review Board.

### Features Used for Clustering

The features that have been shown to be correlated to clinical course or outcomes of COVID-19 were considered. Laboratory values and age were used to identify endotypes (complete list available in Supplementary Material 1). Both the median and the IQR of all lab values for a patient during admission were used as features. Features missing more than 40% of patients were excluded from analysis.

### Variables Used to Examine the Resulting Endotypes

Patient disposition was the primary outcome. Intubation status, length of intubation, length of stay, patient age, race, sex, comorbidities, and treatment with medications commonly used with COVID-19 patients were collected (complete list available in Supplementary Material 2).

### Statistical Analyses

**Figure 1:**
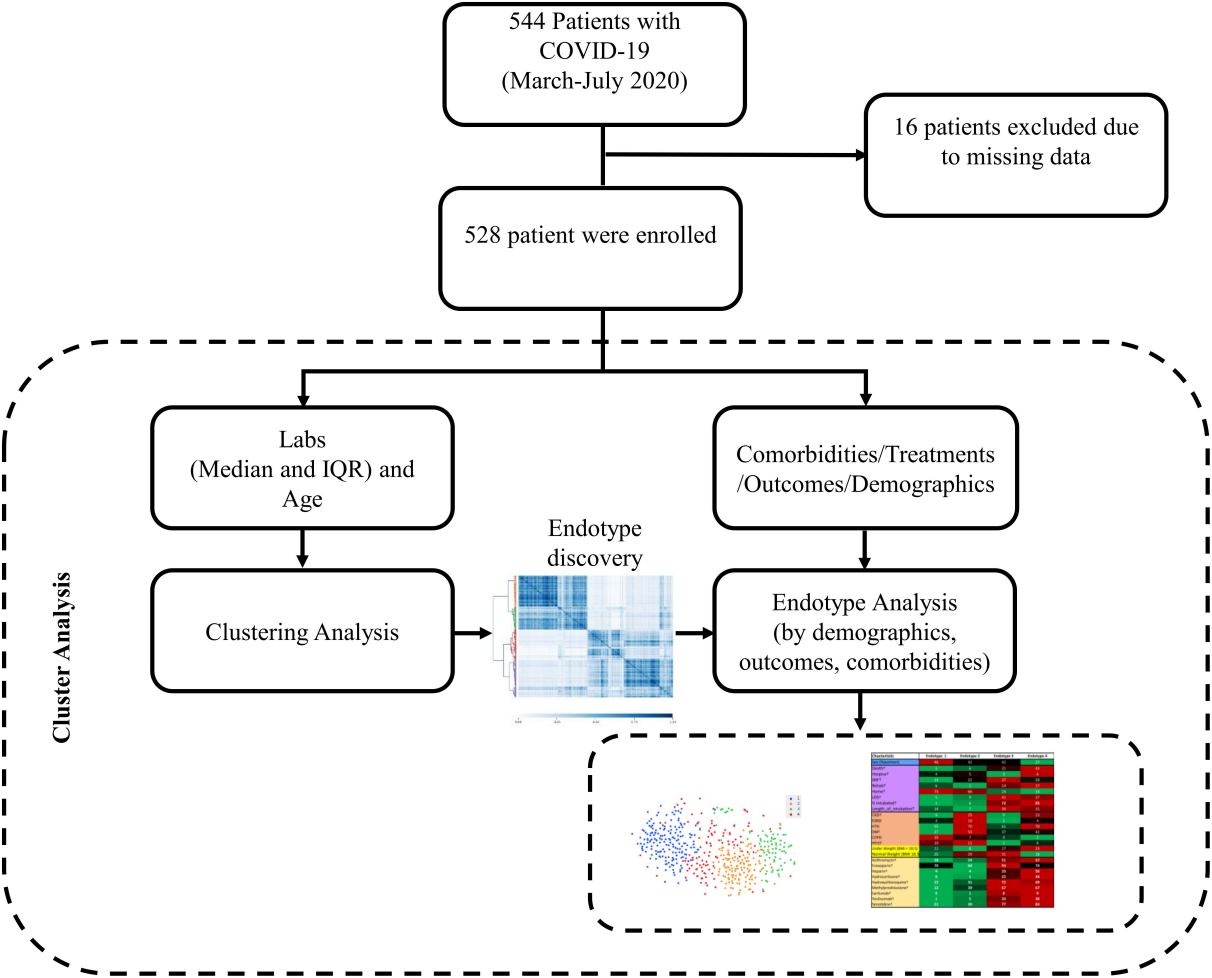
Data collection and analysis schematic. Patients with positive SARS-COV-2 tests that were discharged between March 12, 2020 to July 15, 2020 were included in the study. Labs during hospitalization (median and IQR) and age were the features used for clustering and endotype discovery. Once endotypes were identified, they were analyzed for differences in demographics, outcomes, comorbidities, and treatments.

A schematic presentation of data collection and analysis can be seen in Figure 1. To discover endotypes, we relied on cluster analysis, which generally divides datasets into groups by minimizing the intra-group distance while maximizing the inter-group distance. Instead of using a single clustering algorithm, here we employed ensemble classification (30) by running multiple clustering algorithms (K-mean, Birch, Gaussian Mixture Model, and Agglomerative clustering) and integrating their results. Then, we applied consensus clustering (31) to the results of ensemble classification. Consensus clustering is a robust approach that relies on multiple iterations of the sampled dataset to derive more stable and meaningful clusters and has been widely used to identify biologically meaningful clusters. In our work, the consensus of the ensemble clustering was implemented with 50 bootstraps and 80% of the data.

The stability of consensus matrices (when cluster number K changed from 2 to 10) were measured by obtaining their cumulative distribution function (CDF) as described by Monti et al. (31). Then for each *K* value, proportion of increase in area under the CDF (ΔK), Calinski Harabasz score (CH) (32) and Davies Bouldin score (DBS) (33) were calculated and compared to determine the optimal number of clusters. Finally, to visualize the underlying structure of the data, we generated the data dendrograms by applying hierarchical clustering on the consensus matrices. Pseudocode of our clustering approach is provided in Supplementary Material 3.

To compare the differences between endotypes, the Kruskal-Wallis test (34) and Dunn's multiple comparison test (35) were used for continuous variables, and chi-square tests were used for categorical variables. A significant *p*-value was defined as <0.05. The analysis was performed in MATLAB™ (The Math Works, Inc., Natick, MA) and Python (www.python.org) where we used Opensemble library (36) to perform the consensus clustering.

## Results

Five hundred forty-four patients were identified prospectively for inclusion in the study. Sixteen patients were missing all laboratory data and therefore were excluded from analysis, leaving 528 patients in the final cohort. Baseline characteristics of the final cohort, their comorbidities and hospital characterizations are outlined in Table 1. In the study cohort, the median age was 66 (IQR 55-74), 209 (40%) were female, 103 (19.5%) were African American or Black, 1 (0.2%) was American Indian or Alaska Nation, 7 (1.3%) were Asian, 2 (0.4%) were Native Hawaiian or Other Pacific Islander, 179 (33.9%) were other combinations not described, 119 (22.5%) were White, and 117 (22.5%) declined to specify race. In the cohort, 223 (42.2%) were discharged home, 54 (10.2%) were discharged to rehab, 129 (24.4%) were discharged to a skilled nursing facility, 25 (4.7%) were discharged to hospice, and 97 (18.4%) died in the hospital. Comorbid CKD, ESRD, HTN, and DM were higher in endotype 2 and 4. Length of stay was a median of five days in endotype 1, nine days in endotype 2, 41 days in endotype 3 and 37 days in endotype 4. Percent of patients intubated was 1% in endotype 1, 6% in endotype 2, 72% in endotype 3, and 85% in endotype 4.

**Table 1:** Baseline characteristics of study cohort.

**Characteristic**	**Cohort**
Age – median (IQR)	66 (55–74)
Sex – *n* (%) women	209 (40)
**Race**	
African American or Black– *n* (%)	103 (19.5)
American Indian or Alaska Nation – *n* (%)	1 (0.2)
Asian – *n* (%)	7 (1.3)
Native Hawaiian or Other Pacific Islander– *n* (%)	2 (0.4)
Other combinations not described – *n* (%)	179 (33.9)
White – *n* (%)	119 (22.5)
Declined – *n* (%)	117 (22.2)
**Ethnicity**	
Not Hispanic or Latino or Spanish origin – *n* (%)	147 (27.8)
Latino or Spanish origin – *n* (%)	262 (49.6)
Declined – *n* (%)	119 (22.5)
**Comorbidity**	
CKD (not ESRD) – *n* (%)	81 (15.3)
ESRD– *n* (%)	20 (3.8)
Hypertension– *n* (%)	334 (63.3)
Diabetes Mellitus – *n* (%)	204 (38.6)
Asthma– *n* (%)	41 (7.8)
COPD– *n* (%)	30 (5.7)
Hyperlipidemia– *n* (%)	154 (29.2)
History of stroke– *n* (%)	38 (7.2)
HIV infection– *n* (%)	14 (2.7)
Heart failure–* n* (%)	68 (12.9)
Preserved ejection fraction– *n* (%)	27 (5.1)
Reduced ejection fraction– *n* (%)	34 (6.4)
Unknown ejection fraction– *n* (%)	7 (1.3)
CAD– *n* (%)	74 (14.0)
Obesity (BMI > 30) – *n* (%)	144 (27.3)
**Discharge outcome**	
Home – *n* (%)	223 (42.2)
Rehab – *n* (%)	54 (10.2)
Skilled nursing facility – *n* (%)	129 (24.4)
Hospice – *n* (%)	25 (4.7)
Death – *n* (%)	97 (18.4)
**Hospitalization characteristics**	
Intubated – *n* (%)	234 (44.3)
Days intubated – median (IQR)	31 (17–44)
Length of stay, days – *n* (IQR)	19 (7–44)

### Endotype Descriptions

Features missing in more than 40% of patients were excluded from further analysis: blood pH, blood pCO_2_, blood pO_2_, β-d-Glucan, ionized calcium, and fibrinogen. After considering cluster quality and stability by examining CDF plot, measured ΔK, CH, DBS, and the underlying structure of the data using dendrograms (Supplementary Material 4), we opted for K = 4 which identified four endotypes.

**Figure 2:**
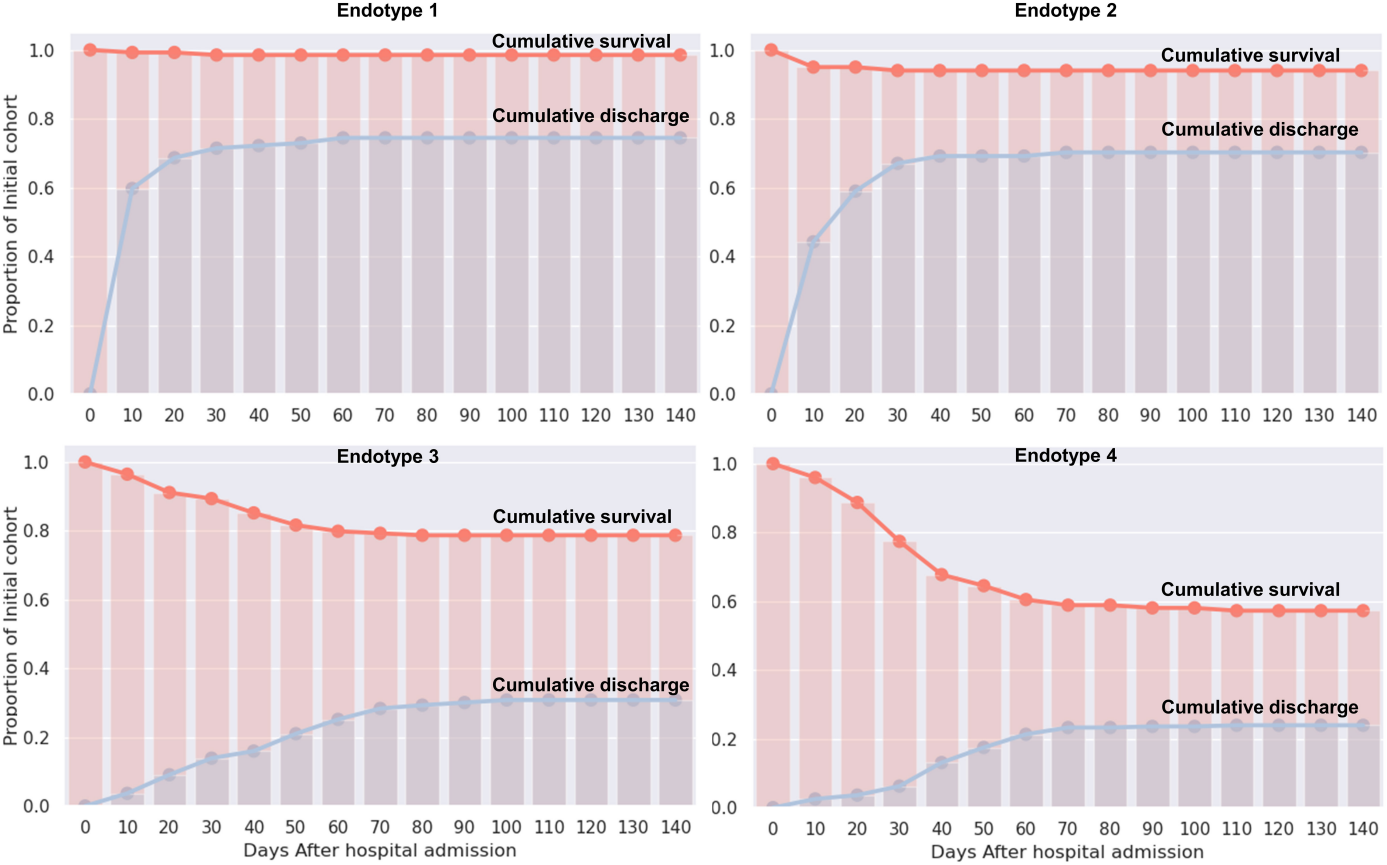
Cumulative survival and discharge out of hospital by endotype. For each endotype, the cumulative survival and cumulative discharge of surviving patients from hospital is displayed in days from admission.

**Figure 3:**
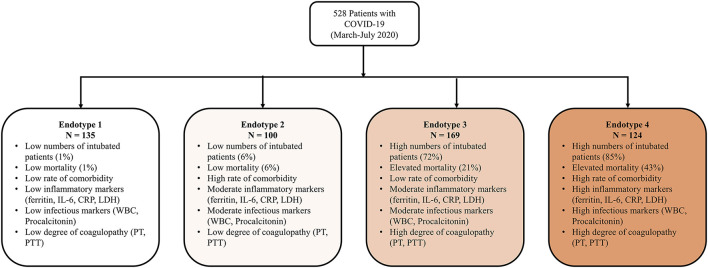
Summary of the four endotypes. A summary of the four identified endotypes is shown.

Median values of the clustering features for each of the four endotypes are outlined in Table 2, Supplementary Material 5. All of the features were significantly different over the endotypes except for median bilirubin and age (*p* > 0.05). Characteristics of the endotypes are outlined in Table 3. Some comorbidities varied significantly across endotypes (i.e., CKD, ESRD, HTN, DM, COPD, heart failure with reduced ejection fraction [HFrEF], and obesity), while others (asthma, hyperlipidemia, HIV infection, history of stroke, heart failure with preserved ejection fraction, and heart failure with unknown EF) did not differ significantly. Treatments differed by endotype (*p* < 0.05) except for remdesivir and prednisone. Mortality and discharge from hospital rates also varied by endotype (Figure 2). Paired comparisons of characteristics are provided in Supplementary Material 6. A summary of the four endotypes is shown in Figure 3.

**Table 2:** Selected endotype features.

Feature^*^	**Endotype 1**	**Endotype 2**	**Endotype 3**	**Endotype 4**
Ferritin_median^†^	219	621	818	1,158
Ferritin_IQR^†^	86	173	455	572
IL6_median^†^	15	35	68	99
IL6_IQR^†^	24	41	92	107
CRPHighSens_median^†^	17	106	107	121
CRPHighSens_IQR^†^	29	84	122	122
ESR_median^†^	38	77	78	64
ESR_IQR^†^	11	22	33	31
LDH_median^†^	287	383	393	498
LDH_IQR^†^	71	102	144	189
WBC_median^†^	7	9	11	12
WBC_IQR^†^	2	2	5	6
NLR_median^†^	3	5	8	12
Hemoglobin_median^†^	12	12	9	8
RDW_median^†^	14	14	15	16
Procalcitonin_median^†^	0.1	0.3	0.4	1.4
Procalcitonin_IQR^†^	0.1	0.1	0.4	2.5
Platelet_median^†^	223	262	283	209
Platelet_IQR^†^	39	64	113	113
DDimer_median^†^	0.9	1.7	3.6	5.3
Prothrombin_median^†^	14.2	14.6	15.1	15.5
Prothrombin_IQR^†^	0.9	0.7	1.2	1.6
PTT_median^†^	32	33	36	44
hsTnT_median^†^	16	26	25	62
CK_total_median	89	115	112	138
BNP_median^†^	298	931	1,026	2,702
Lactate_median^†^	1.5	1.7	1.4	1.4
CO2_median^†^	24	23	26	22
BUN_median^†^	15	25	24	48
Creatinine_median^†^	0.9	1.0	0.8	2.4
Glucose_median^†^	107	138	144	144
Calcium_median^†^	9	9	8	8
Phosphorus_median^†^	3.5	3.5	3.2	4.1
AST_median^†^	28	39	35	46
ALT_median^†^	21	31	35	33
Alkphos_median^†^	77	90	96	109
Bilirubin_direct_median^†^	0.1	0.2	0.2	0.3
TotalProtein_median^†^	6.7	6.5	6.0	5.8
albumin_median^†^	4	3	3	3

* *Values are color-coded for each feature from red (high) to black (median) to green (low). Full set of features can be found in Supplementary Material 5*.

† *Denotes p < 0.001*.

**Table 3:** Endotype characteristics.

Characteristic^*^	**Endotype 1**	**Endotype 2**	**Endotype 3**	**Endotype 4**
Patients, *n* (%)	135 (26)	100 (19)	169 (32)	124 (23)
Age, median years ^‡^	68.0	67.5	66.0	64.0
Sex, % women	46	42	42	27
Death, %^†^	1	6	21	43
Hospice, %^†^	4	5	3	6
SNF, %^†^	14	22	37	20
Rehab, %^†^	6	1	14	17
Home, %^†^	73	66	24	14
LOS, median days^†^	5	9	41	37
Intubated, %^†^	1	6	72	85
Intubated, median days^†^	14	7	34	31
CKD, %^†^	9	25	9	23
ESRD, %	3	10	1	4
HTN, %	55	70	61	70
DM, %^†^	27	53	37	42
COPD, %	10	7	4	2
HFrEF	10	11	1	6
Obesity (BMI > 30), %	27	22	25	35
Azithromycin, %^†^	18	24	51	57
Enoxaparin, %^†^	70	64	93	70
Heparin, %^†^	4	4	33	56
Hydrocortisone, %^†^	0	1	25	44
Hydroxychloroquine, %^†^	13	31	72	69
Methylprednisolone, %^†^	12	39	67	67
Sarilumab, %^†^	0	1	8	9
Tocilizumab, %^†^	1	5	24	30
Famotidine, %^†^	21	30	77	83

* *Characteristics are only shown if p < 0.05 indicating that values of the four groups are significantly different. Values are color-coded by characteristic from red (high) to black (median) to green (low). Number of patients included for reference, but no statistical test was run*.

† *Denotes p < 0.001*.

‡ *Age included for reference in this table but was a feature used in clustering and p > 0.05*.

Endotype 1 patients had a median age of 68 years, had the most women (46%), the lowest prevalence of mortality (1%), shortest hospital length-of-stay (median: 5 days), and fewest intubated patients (1%). This endotype had the lowest prevalence of HTN and DM and greatest prevalence of COPD. Endotype 1 patients had the lowest inflammatory markers (ferritin, IL-6, CRP, ESR, LDH), lowest infectious markers (WBC, procalcitonin), and lowest degree of coagulopathy (PT and PTT, but not significantly < endotype 2). Endotype 1 patients received the least of any endotype of the reviewed medications (except for enoxaparin) but overall had similar medication use as endotype 2 (except for hydroxychloroquine and methylprednisolone).

Endotype 2 patients had a median age of 67.5 years, included approximately the cohort average of women (42%), second-lowest mortality (6%), relatively short hospital length-of-stay (median: 9 days), and second-fewest intubations (6%). This patient subgroup had the most comorbidities (CKD, ESRD, HTN, DM, and HFrEF). Endotype 2 patients had similar inflammatory markers to endotype 3 (ferritin, CRP, ESR, and LDH but not IL-6 which was significantly lower than endotype 3), second lowest infectious markers (WBC and procalcitonin, although procalcitonin was not significantly < endotype 3), and second least degree of coagulopathy (PT and PTT, but not significantly more than endotype 1). Endotype 2 patients received less of the reviewed medications than endotypes 3 and 4 except for enoxaparin which was not significantly < endotype 4.

Endotype 3 patients had a median age of 66 years, included approximately the cohort average of women (42%), exhibited a mortality of 21%, had the longest hospital length-of-stay (median: 41 days), and had the second-highest prevalence of intubation (72%). Patients in this endotype had a relatively low number of comorbidities. Endotype 3 patients had similar inflammatory markers as endotype 2 (ferritin, CRP, ESR, and LDH, but not IL-6 which was significantly higher), second-highest infectious markers (WBC and procalcitonin, although procalcitonin was not significantly > endotype 2), and second-highest coagulopathy markers (PT and PTT, but PT was not significantly < endotype 4). Endotype 3 patients received reviewed medications at similar rates as patients in endotype 4 (except for enoxaparin, heparin, and hydrocortisone).

Endotype 4 patients had a median age of 64 years, included the fewest women (27%), greatest degree of mortality (43%), a fairly long hospital length-of-stay (median 37 days), and were the most intubated (85%). This endotype had moderate amounts of CKD and ESRD, higher amounts of HTN, and the most obesity. Endotype 4 patients had the highest inflammatory markers (ferritin, LDH were significantly higher than endotype 3 while IL-6 and CRP were similarly high as endotype 3), highest infectious markers (WBC, procalcitonin), and greatest degree of coagulopathy (PT and PTT, but PT was not significantly > endotype 3). The exception was ESR which was lower than endotypes 2 and 3. Endotype 4 patients received the most of the reviewed medications (except for enoxaparin and hydroxychloroquine). Of the medications, only hydrocortisone and heparin use were significantly more than in endotype 3.

## Discussion

Our study has three main findings: first, four distinct groups of patients were identified though consensus clustering of ensemble classification using age and laboratory values over the entire hospitalization as features. The groups as a whole did not vary significantly by age or race but had differences in sex as well as comorbidities. We consider these patient subgroups to comprise endotypes (14) since the data used to segregate them include variables that are indicative of physiologic and inflammatory dysfunction. The endotypes were also treated with differing medications in the hospital. Endotype 1 and 2 exhibited low mortality and short length of stay. However, Endotype 2 had slightly worse outcomes and slightly higher inflammatory and organ damage markers. Endotypes 3 and 4 had more mortality and length of stay, with endotype 4 having a markedly high mortality at 43% and the highest levels markers of inflammation and end-organ dysfunction.

Second, we identified endotypes of COVID-19 patients with widely disparate outcomes that were not expected based on classic risk factors such as age, sex, and preexisting comorbidities (3, 6). We documented patients with lower-risk features who had worse courses than traditionally expected. Endotype 2 had the greatest number of comorbidities overall but a relatively low mortality. Focusing on comorbidities alone would have resulted in misclassification of endotype 2 patients. Along the same lines, endotype 3 had many fewer comorbidities than endotype 2, and yet endotype 3 had significantly worse outcomes. IL-6, d-dimer, and WBC are significantly higher in endotype 3 compared to endotype 2. Further examination of the different endotypes has potential to yield clinical and pathobiological insight into what is driving the vastly different clinical courses experienced by patients with COVID-19.

Third, consensus clustering of ensemble classification (37) supported the previously hypothesized existence of subgroups of COVID-19 manifestations. In part because elevated inflammatory markers such as C-reactive protein, ferritin, and IL-6 were associated with poor outcomes (38, 39), steroids were studied and proven effective at treating severe COVID-19 (5). Patients meeting a proposed criteria for COVID-19-associated hyperinflammatory syndrome (including fever; ferritin and d-dimer elevation; NLR elevation or anemia/thrombocytopenia; LDH or AST elevation; and IL-6, triglyceride, or CRP elevation) were shown recently to have higher risk of requiring mechanical ventilation and higher risk of mortality (13). The endotypes we identified that have higher levels of circulating inflammatory markers have worse outcomes than patient clusters with lower inflammatory markers. This appears to hold true even when patients are intubated, such as in endotypes 3 and 4 in which a higher number of patients were intubated, but where there were notably higher mortality and inflammatory markers in endotype 4. Endotype 4 patients also had notably higher procalcitonin levels, a potential indication that these patients with higher inflammatory markers may have experienced more (or more severe) bacterial infections.

Identification of endotypes has several potential useful functions. Endotypes may point to unique pathobiologic mechanisms of disease that warrant further investigation in each specific subset of patients. Different endotypes may respond differently to treatments and may explain the heterogeneity of disease course. Examining endotypes for differential response to treatments could identify subsets of patients where treatments are beneficial. If endotypes can be identified early in disease course, endotypes can offer prognostic and clinical management information. Future studies will need to validate these endotypes.

There are several limitations to our study. First, this is a single-center study that prospectively collected data from patients admitted to telemetry capable beds. We have not validated the endotypes in the setting of more recent SARS-COV-2 variants. However, in the setting of this fast-moving disease, validation of endotypes in the setting of the most recent variant will continue to be a challenge for any large COVID-19 cohort study. Second, there were some lab variables with a high amount of missing data. These variables were dropped which may have introduced some bias. Third, standard of care treatments for patients with COVID-19 changed over time. The treatments each endotype received may have been changing over time. Dosing data for medications was not available, therefore anticoagulation medications were not classified as prophylactic or therapeutic. Fourth, the admission criteria for patients with COVID-19 may have changed over time.

In conclusion, disease endotypes have the potential to describe a subset of patients that are undergoing shared biologic processes resulting in a similar phenotype of disease and may identify groups of patients with different clinical courses and responses to therapy. However, having certain high or low risk features does not guarantee association with a certain outcome; rather, patients with certain features appear to have one of multiple different clinical courses. In this cohort of patients hospitalized with COVID-19, we identified four unique endotypes of patients by using clustering of laboratory values throughout the hospitalization as well as patient age. The endotypes had differences in inflammatory markers, infectious markers, evidence of end-organ dysfunction, comorbidities, and outcomes. Further work is needed to validate these endotypes in other cohorts and study endotype differences to treatment response.

## Data Availability Statement

The original contributions presented in the study are included in the article/Supplementary Materials, further inquiries can be directed to the corresponding author/s.

## Ethics Statement

The studies involving human participants were reviewed and approved by Columbia University. Written informed consent for participation was not required for this study in accordance with the national legislation and the institutional requirements.

## Author Contributions

BR, MM, KT, JC, MP, GC, YV, SA, and SP were involved in the conception and design of the study. BR, MM, KT, KD, MP, GC, YV, SA, and SP were involved in the acquisition and/or analysis. BR, MM, KT, MP, GC, YV, SA, and SP interpreted the data. All authors were involved in drafting the manuscript or critical appraisal and approved the final version of the manuscript.

## Funding

The following grants supported this study: NIH R21 NS113055 (SP), AHA 20POST35210653 (MM), and NIH U01EB021960 (YV).

## Conflict of Interest

YV is a co-founder of, and stakeholder in Immunetrics, Inc. The remaining authors declare that the research was conducted in the absence of any commercial or financial relationships that could be construed as a potential conflict of interest.

## Publisher's Note

All claims expressed in this article are solely those of the authors and do not necessarily represent those of their affiliated organizations, or those of the publisher, the editors and the reviewers. Any product that may be evaluated in this article, or claim that may be made by its manufacturer, is not guaranteed or endorsed by the publisher.
