# Supplementary material for: Identification of Endotypes of Hospitalized COVID-19 Patients
Source: Front Med (Lausanne). 2021 Nov 11;8:770343. doi: 10.3389/fmed.2021.770343 (PMC8632028; doi:10.3389/fmed.2021.770343)
Supplement: Supplementary file 1 [file Data_Sheet_1.DOCX]

**Online Data Supplement**

**Contents**

[S1 Features used for clustering 2](#_Toc81552384)

[S2 Variables used to examine the resulting endotypes 3](#_Toc81552385)

[S3 Pseudocode for consensus clustering 4](#_Toc81552386)

[S4: Consensus clustering analysis of the COVID-19 features 5](#_Toc81552387)

[S5: Endotype features 6](#_Toc81552388)

[S6: Paired comparisons of features and outcomes by endotype pairs 8](#_Toc81552389)

# S1 Features used for clustering

The features that have been shown to be correlated to clinical course or outcomes of COVID-19 were considered. Laboratory values and age were used to identify endotypes. Given that most common laboratories on admission had literature supporting some correlation (at least weak) to COVID-19 clinical course, all laboratory values typically obtained on an admitted patient were included, as were some laboratory values that were only checked commonly on patients with COVID-19: common chemistries (sodium, potassium, chloride, bicarbonate (CO2), blood urea nitrogen [BUN], creatinine, glucose, calcium, magnesium, phosphorus, aspartate transaminase [AST], alanine aminotransferase [ALT], alkaline phosphatase [ALP], bilirubin total, bilirubin direct, total protein, albumin); hematologic labs (white blood cell count [WBC], hemoglobin [Hgb], platelets [plt], lymphocyte percent, neutrophil percent, neutrophil to lymphocyte ratio [NLR], red cell distribution width [RDW]; coagulation measures (partial thromboplastin time [PTT], prothrombin time [PT], d-dimer); inflammatory markers (erythrocyte sedimentation rate [ESR], high sensitivity C-reactive protein [hs-CRP], ferritin, interleukin-6 [IL-6], lactate dehydrogenase [LDH]); and other labs (procalcitonin, high sensitivity troponin T [hsTnT], N-Terminal-B-type Natriuretic Peptide [BNP], creatinine kinase [CK], lactate [venous or arterial], blood pH, blood pCO2, blood pO2, ionized calcium, β-d-Glucan). Features missing more than 40% of patients were excluded from analysis.

# S2 Variables used to examine the resulting endotypes

Patient disposition was the primary outcome (death, hospice, skilled nursing facility (SNF) or long-term care facility, acute inpatient rehab, or home). Intubation rate, length of intubation, and length of stay were also examined. In order to exclude patients intubated primarily for a procedure, patients were only counted as intubated if intubation lasted at least 24 hours. Race, sex, and comorbidities at admission (obesity, diabetes mellitus, hypertension, chronic kidney disease not on dialysis, end-stage renal disease on dialysis, asthma, COPD, hyperlipidemia, stroke, HIV infection, heart failure (with and without preserved ejection fraction), coronary artery disease (CAD)) were collected for all patients to allow for examination of race, sex, and comorbidities by endotype. We examined the endotypes for exposure to interventions which were unfortunately neither uniform nor stable across time as the scientific community adapted to growing knowledge of optimal treatment for COVID. The interventions of interest included: azithromycin, hydroxychloroquine, remdesivir, methylprednisolone, hydrocortisone, prednisone, sarilumab, tocilizumab, famotidine, heparin drip, and enoxaparin.

# S3 Pseudocode for consensus clustering

***Algorithm 1:*** *Consensus clustering*

**input:** *data (feature matrix)*

**output:** *consensus_matrix_k*

**for** *k = 2:10*

*consensus_matrx* = []

**for** *n=1:50*

data_sample = sample(data,0.8)

algorithms = {k-means,Birch,GMM,Agglomerative,Spectral}

ensemble_c = cluster(data_sample, algorithms)

consesus_matrix(n)= ensemble_C

c_final_k = Merge(consesus_matrix)

**consensus_matrix_k(**k**) =** c_final_k

# S4: Consensus clustering analysis of the COVID-19 features


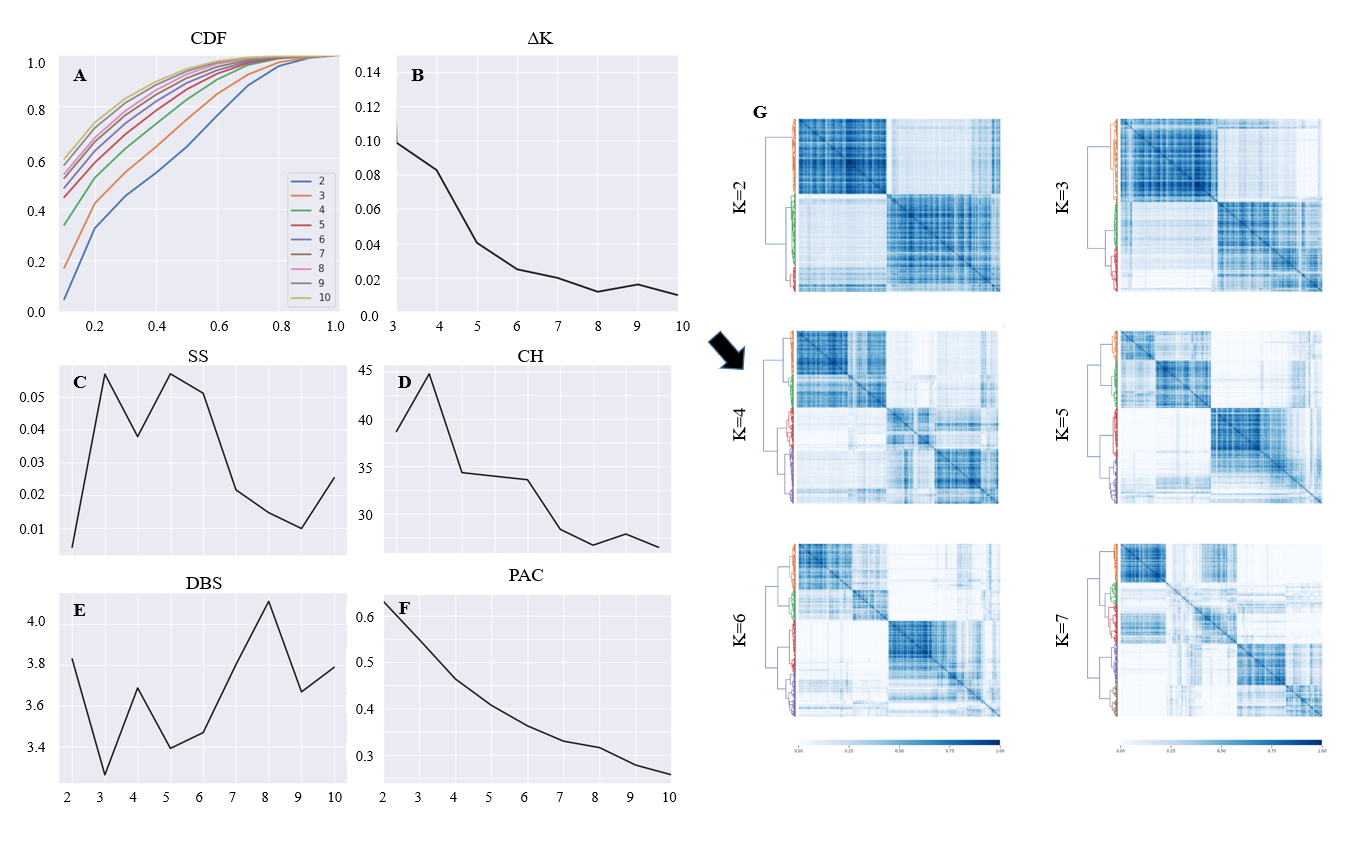
(A) Cumulative Distribution function plot for varying K; (B) change in area under the CDF plots indicating information gain drops off after K = 4 (C) Silhouette Score (SS) (D) Clainski Harabrasz (CH) score (E) Davies Bouldin Score (DBS) and (F) proportion of ambiguous clustering (PAC) score with K varied from 2 to 10. (G) Consensus matrix for K = 2, …, 7 represented as heatmaps. Patients’ samples are both rows and columns and consensus values range from 0 (never clustered together) to 1 (always clustered together), the arrow at K = 4 highlights the most stable cluster.

# S5: Endotype features

The median values of features for the discovered four endotypes. Features are only shown if p < 0.05 indicating that values of the four groups are significantly different. Values are color-coded for each feature from red (high) to black (median) to green (low). †denotes p < 0.001

| **Feature** | **Endotype 1** | **Endotype 2** | **Endotype 3** | **Endotype 4** |
| --- | --- | --- | --- | --- |
| Ferritin_median† | **219** | **621** | **818** | **1158** |
| Ferritin_IQR† | **86** | **173** | **455** | **572** |
| IL6_median† | **15** | **35** | **68** | **99** |
| IL6_IQR† | **24** | **41** | **92** | **107** |
| CRPHighSens_median† | **17** | **106** | **107** | **121** |
| CRPHighSens_IQR† | **29** | **84** | **122** | **122** |
| ESR_median† | **38** | **77** | **78** | **64** |
| ESR_IQR† | **11** | **22** | **33** | **31** |
| LDH_median† | **287** | **383** | **393** | **498** |
| LDH_IQR† | **71** | **102** | **144** | **189** |
| WBC_median† | **7** | **9** | **11** | **12** |
| WBC_IQR† | **2** | **2** | **5** | **6** |
| LymphocytePct_median† | **20** | **14** | **10** | **7** |
| LymphocytePct_IQR† | **7** | **5** | **7** | **6** |
| Lymphocyte_median† | **1.4** | **1.1** | **1.1** | **1.0** |
| Lymphocyte_IQR† | **0.4** | **0.3** | **0.6** | **0.6** |
| Neutrophil_median† | **5** | **6** | **8** | **10** |
| Neutrophil_IQR† | **1.3** | **2.2** | **4.9** | **5.4** |
| NLR_median† | **3** | **5** | **8** | **12** |
| NLR_IQR† | **2** | **3** | **7** | **10** |
| Hemoglobin_median† | **12** | **12** | **9** | **8** |
| Hemoglobin_IQR† | **0.8** | **1.0** | **1.7** | **1.6** |
| RDW_median† | **14** | **14** | **15** | **16** |
| RDW_IQR† | **0.3** | **0.4** | **1.4** | **1.7** |
| Procalcitonin_median† | **0.1** | **0.3** | **0.4** | **1.4** |
| Procalcitonin_IQR† | **0.1** | **0.1** | **0.4** | **2.5** |
| Platelet_median† | **223** | **262** | **283** | **209** |
| Platelet_IQR† | **39** | **64** | **113** | **113** |
| DDimer_median† | **0.9** | **1.7** | **3.6** | **5.3** |
| DDimer_IQR† | **0.3** | **0.8** | **2.9** | **6.4** |
| Prothrombin_median† | **14.2** | **14.6** | **15.1** | **15.5** |
| Prothrombin_IQR† | **0.9** | **0.7** | **1.2** | **1.6** |
| PTT_median† | **32** | **33** | **36** | **44** |
| PTT_IQR† | **4** | **3** | **8** | **27** |
| hsTnT_median† | **16** | **26** | **25** | **62** |
| hsTNT_IQR† | **3** | **6** | **16** | **51** |
| CK_total_median | **89** | **115** | **112** | **138** |
| CK_total_IQR† | **46** | **122** | **126** | **230** |
| BNP_median† | **298** | **931** | **1026** | **2702** |
| BNP_IQR† | **858** | **463** | **525** | **2771** |
| Lactate_median† | **1.5** | **1.7** | **1.4** | **1.4** |
| Lactate_IQR† | **0.7** | **0.7** | **0.7** | **0.8** |
| Sodium_median† | **140** | **139** | **142** | **140** |
| Sodium_IQR† | **2.5** | **3.0** | **6.0** | **5.0** |
| Potassium_median† | **4.2** | **4.5** | **4.2** | **4.4** |
| Potassium_IQR† | **0.5** | **0.5** | **0.7** | **0.7** |
| Chloride_median | **101** | **101** | **103** | **102** |
| Chloride_IQR† | **3** | **4** | **6** | **6** |
| CO2_median† | **24** | **23** | **26** | **22** |
| CO2_IQR† | **2.3** | **3.0** | **4.5** | **4.0** |
| BUN_median† | **15** | **25** | **24** | **48** |
| BUN_IQR† | **4** | **8** | **14** | **35** |
| Creatinine_median† | **0.9** | **1.0** | **0.8** | **2.4** |
| Creatinine_IQR† | **0.1** | **0.2** | **0.4** | **1.9** |
| Glucose_median† | **107** | **138** | **144** | **144** |
| Glucose_IQR† | **26** | **50** | **57** | **57** |
| Calcium_median† | **9** | **9** | **8** | **8** |
| Calcium_IQR† | **0.4** | **0.4** | **0.6** | **0.7** |
| Magnesium_median† | **2.1** | **2.2** | **2.1** | **2.4** |
| Magnesium_IQR† | **0.2** | **0.2** | **0.3** | **0.4** |
| Phosphorus_median† | **3.5** | **3.5** | **3.2** | **4.1** |
| Phosphorus_IQR† | **0.5** | **0.6** | **1.0** | **1.9** |
| AST_median† | **28** | **39** | **35** | **46** |
| AST_IQR† | **9** | **16** | **23** | **30** |
| ALT_median† | **21** | **31** | **35** | **33** |
| ALT_IQR† | **5** | **11** | **24** | **27** |
| Alkphos_median† | **77** | **90** | **96** | **109** |
| Alkphos_IQR† | **8** | **14** | **31** | **41** |
| Bilirubin_IQR† | **0.2** | **0.1** | **0.2** | **0.3** |
| Bilirubin_direct_median† | **0.1** | **0.2** | **0.2** | **0.3** |
| Bilirubin_direct_IQR† | **0.1** | **0.1** | **0.2** | **0.3** |
| TotalProtein_median† | **6.7** | **6.5** | **6.0** | **5.8** |
| TotalProtein_IQR† | **0.5** | **0.5** | **0.8** | **0.8** |
| albumin_median† | **4** | **3** | **3** | **3** |
| albumin_IQR† | **0.4** | **0.3** | **0.5** | **0.5** |

# S6: Paired comparisons of features and outcomes by endotype pairs

Only features or characteristics that were significantly different across all endotypes were included in the table (p < 0.05 in the Kruskal-Wallis or chi-square tests). For paired comparisons, Dunn's multiple comparison test (34) was used and corrected p-values are displayed.

| Characteristic | Endotype 1 vs 2 | Endotype 1 vs 3 | Endotype 1 vs 4 | Endotype 2 vs 3 | Endotype 2 vs 4 | Endotype 3 vs 4 |
| --- | --- | --- | --- | --- | --- | --- |
| Demographics |  |  |  |  |  |  |
| Age | 1.000 | 1.000 | 0.260 | 1.000 | 0.099 | 0.810 |
| Sex | 0.769 | 0.769 | 0.019 | 0.900 | 0.063 | 0.043 |
| Outcome |  |  |  |  |  |  |
| Death | 0.127 | <0.0005 | <0.0005 | 0.002 | <0.0005 | <0.0005 |
| Hospice | 0.865 | 0.865 | 0.865 | 0.865 | 0.865 | 0.865 |
| SNF | 0.238 | <0.0005 | 0.306 | 0.027 | 0.864 | 0.007 |
| Rehab | 0.131 | 0.048 | 0.018 | 0.002 | 0.001 | 0.633 |
| Home | 0.284 | <0.0005 | <0.0005 | <0.0005 | <0.0005 | 0.044 |
| LOS | 0.093 | <0.0005 | <0.0005 | <0.0005 | <0.0005 | 1.000 |
| Intubated, % | 0.050 | <0.0005 | <0.0005 | <0.0005 | <0.0005 | 0.009 |
| Intubation, days | 1.000 | <0.0005 | <0.0005 | <0.0005 | <0.0005 | 0.211 |
| Comorbidity |  |  |  |  |  |  |
| CKD | 0.005 | 0.979 | 0.006 | 0.005 | 0.949 | 0.006 |
| ESRD | 0.145 | 0.295 | 0.897 | 0.003 | 0.198 | 0.198 |
| HTN | 0.078 | 0.404 | 0.078 | 0.258 | 0.904 | 0.258 |
| DM | <0.0005 | 0.099 | 0.034 | 0.034 | 0.156 | 0.493 |
| COPD | 0.507 | 0.172 | 0.046 | 0.507 | 0.179 | 0.507 |
| HFrEF | 0.900 | 0.006 | 0.589 | 0.005 | 0.509 | 0.064 |
| Obesity (BMI > 30) | 0.454 | 0.781 | 0.232 | 0.570 | 0.057 | 0.111 |
| Medications |  |  |  |  |  |  |
| Azithromycin | 0.336 | <0.0005 | <0.0005 | <0.0005 | <0.0005 | 0.336 |
| Enoxaparin | 0.531 | <0.0005 | 0.966 | <0.0005 | 0.531 | <0.0005 |
| Heparin | 0.821 | <0.0005 | <0.0005 | <0.0005 | <0.0005 | <0.0005 |
| Hydrocortisone | 0.880 | <0.0005 | <0.0005 | <0.0005 | <0.0005 | 0.002 |
| Hydroxychloroquine | 0.002 | <0.0005 | <0.0005 | <0.0005 | <0.0005 | 0.691 |
| Methylprednisolone | <0.0005 | <0.0005 | <0.0005 | <0.0005 | <0.0005 | 0.910 |
| Sarilumab | 0.973 | 0.005 | 0.005 | 0.038 | 0.038 | 0.973 |
| Tocilizumab | 0.285 | <0.0005 | <0.0005 | <0.0005 | <0.0005 | 0.350 |
| Famotidine | 0.218 | <0.0005 | <0.0005 | <0.0005 | <0.0005 | 0.254 |
| Laboratory Values |  |  |  |  |  |  |
| Ferritin_median | <0.0005 | <0.0005 | <0.0005 | 0.431 | <0.0005 | 0.046 |
| Ferritin_IQR | 0.001 | <0.0005 | <0.0005 | <0.0005 | <0.0005 | 0.988 |
| IL6_median | 0.003 | <0.0005 | <0.0005 | <0.0005 | <0.0005 | 0.256 |
| IL6_IQR | 0.073 | <0.0005 | <0.0005 | <0.0005 | <0.0005 | 0.716 |
| CRPHighSens_median | <0.0005 | <0.0005 | <0.0005 | 1.000 | 0.481 | 1.000 |
| CRPHighSens_IQR | <0.0005 | <0.0005 | <0.0005 | <0.0005 | <0.0005 | 1.000 |
| ESR_median | <0.0005 | <0.0005 | <0.0005 | 1.000 | 0.056 | 0.001 |
| ESR_IQR | 0.161 | <0.0005 | <0.0005 | <0.0005 | <0.0005 | 1.000 |
| LDH_median | <0.0005 | <0.0005 | <0.0005 | 1.000 | <0.0005 | <0.0005 |
| LDH_IQR | 0.113 | <0.0005 | <0.0005 | <0.0005 | <0.0005 | 0.084 |
| WBC_median | 0.016 | <0.0005 | <0.0005 | <0.0005 | <0.0005 | 0.012 |
| WBC_IQR | 0.001 | <0.0005 | <0.0005 | <0.0005 | <0.0005 | 0.009 |
| Lymphocyte %_median | <0.0005 | <0.0005 | <0.0005 | <0.0005 | <0.0005 | 0.007 |
| Lymphocyte %_IQR | 1.000 | <0.0005 | 0.002 | <0.0005 | 0.254 | 0.119 |
| Lymphocyte_median | 0.003 | <0.0005 | <0.0005 | 1.000 | 0.195 | 0.088 |
| Lymphocyte_IQR | 0.532 | <0.0005 | <0.0005 | <0.0005 | <0.0005 | 1.000 |
| Neutrophil_median | 0.015 | <0.0005 | <0.0005 | <0.0005 | <0.0005 | 0.032 |
| Neutrophil_IQR | 0.002 | <0.0005 | <0.0005 | <0.0005 | <0.0005 | 0.167 |
| NLR_median | <0.0005 | <0.0005 | <0.0005 | <0.0005 | <0.0005 | 0.014 |
| NLR_IQR | <0.0005 | <0.0005 | <0.0005 | <0.0005 | <0.0005 | 0.013 |
| Hemoglobin_median | 0.324 | <0.0005 | <0.0005 | <0.0005 | <0.0005 | <0.0005 |
| Hemoglobin_IQR | 0.042 | <0.0005 | <0.0005 | <0.0005 | <0.0005 | 1.000 |
| RDW_median | 1.000 | <0.0005 | <0.0005 | <0.0005 | <0.0005 | 0.010 |
| RDW_IQR | 0.978 | <0.0005 | <0.0005 | <0.0005 | <0.0005 | 0.606 |
| Procalcitonin_median | <0.0005 | <0.0005 | <0.0005 | 1.000 | <0.0005 | <0.0005 |
| Procalcitonin_IQR | 0.025 | <0.0005 | <0.0005 | <0.0005 | <0.0005 | <0.0005 |
| Platelet_median | 0.032 | <0.0005 | 0.490 | 1.000 | <0.0005 | <0.0005 |
| Platelet_IQR | <0.0005 | <0.0005 | <0.0005 | <0.0005 | <0.0005 | 1.000 |
| DDimer_median | 0.276 | <0.0005 | <0.0005 | <0.0005 | <0.0005 | 0.006 |
| DDimer_IQR | 0.140 | <0.0005 | <0.0005 | <0.0005 | <0.0005 | 0.026 |
| PT_median | 0.328 | <0.0005 | <0.0005 | 0.016 | <0.0005 | 0.085 |
| PT_IQR | 0.432 | <0.0005 | <0.0005 | <0.0005 | <0.0005 | 0.048 |
| PTT_median | 1.000 | <0.0005 | <0.0005 | 0.016 | <0.0005 | <0.0005 |
| PTT_IQR | 0.724 | <0.0005 | <0.0005 | <0.0005 | <0.0005 | <0.0005 |
| hsTnT_median | 0.012 | 0.003 | <0.0005 | 1.000 | <0.0005 | <0.0005 |
| hsTnT_IQR | 0.516 | <0.0005 | <0.0005 | <0.0005 | <0.0005 | <0.0005 |
| CK_total_median | 0.072 | 1.000 | 0.001 | 0.960 | 1.000 | 0.048 |
| CK_total_IQR | 0.209 | <0.0005 | <0.0005 | <0.0005 | <0.0005 | 0.286 |
| BNP_median | 0.001 | <0.0005 | <0.0005 | 1.000 | 0.066 | 0.198 |
| BNP_IQR | 1.000 | <0.0005 | <0.0005 | <0.0005 | <0.0005 | 0.012 |
| Lactate_median | 0.098 | 0.451 | 0.681 | <0.0005 | 0.001 | 1.000 |
| Lactate_IQR | 0.232 | <0.0005 | <0.0005 | <0.0005 | <0.0005 | 1.000 |
| Sodium_median | 0.141 | <0.0005 | 1.000 | <0.0005 | 0.307 | <0.0005 |
| Sodium_IQR | 0.011 | <0.0005 | <0.0005 | <0.0005 | <0.0005 | 1.000 |
| Potassium_median | <0.0005 | 1.000 | 0.004 | <0.0005 | 1.000 | <0.0005 |
| Potassium_IQR | 0.297 | <0.0005 | <0.0005 | <0.0005 | <0.0005 | 0.033 |
| Chloride_median | 0.593 | 0.136 | 1.000 | 0.001 | 0.086 | 1.000 |
| Chloride_IQR | 0.007 | <0.0005 | <0.0005 | <0.0005 | <0.0005 | 1.000 |
| CO2_median | 0.056 | <0.0005 | <0.0005 | <0.0005 | 0.048 | <0.0005 |
| CO2_IQR | 0.022 | <0.0005 | <0.0005 | <0.0005 | <0.0005 | 1.000 |
| BUN_median | <0.0005 | <0.0005 | <0.0005 | 1.000 | <0.0005 | <0.0005 |
| BUN_IQR | <0.0005 | <0.0005 | <0.0005 | <0.0005 | <0.0005 | <0.0005 |
| Creatinine_median | 0.014 | 1.000 | <0.0005 | <0.0005 | <0.0005 | <0.0005 |
| Creatinine_IQR | <0.0005 | <0.0005 | <0.0005 | 0.006 | <0.0005 | <0.0005 |
| Glucose_median | <0.0005 | <0.0005 | <0.0005 | 0.554 | 1.000 | 1.000 |
| Glucose_IQR | <0.0005 | <0.0005 | <0.0005 | 1.000 | 1.000 | 1.000 |
| Calcium_median | 0.002 | <0.0005 | <0.0005 | 0.019 | <0.0005 | 0.037 |
| Calcium_IQR | 1.000 | <0.0005 | <0.0005 | <0.0005 | <0.0005 | 0.425 |
| Magnesium_median | 0.010 | 0.196 | <0.0005 | 1.000 | <0.0005 | <0.0005 |
| Magnesium_IQR | 0.954 | <0.0005 | <0.0005 | <0.0005 | <0.0005 | 0.001 |
| Phosphorus_median | 0.134 | 1.000 | <0.0005 | 0.011 | 0.001 | <0.0005 |
| Phosphorus_IQR | 0.105 | <0.0005 | <0.0005 | <0.0005 | <0.0005 | <0.0005 |
| AST_median | <0.0005 | <0.0005 | <0.0005 | 1.000 | 0.429 | 0.003 |
| AST_IQR | <0.0005 | <0.0005 | <0.0005 | 0.001 | <0.0005 | 0.052 |
| ALT_median | <0.0005 | <0.0005 | <0.0005 | 1.000 | 1.000 | 1.000 |
| ALT_IQR | <0.0005 | <0.0005 | <0.0005 | <0.0005 | <0.0005 | 1.000 |
| Alkphos_median | 0.005 | <0.0005 | <0.0005 | 1.000 | 0.006 | 0.105 |
| Alkphos_IQR | <0.0005 | <0.0005 | <0.0005 | <0.0005 | <0.0005 | 0.190 |
| Bilirubin_IQR | 0.059 | <0.0005 | <0.0005 | <0.0005 | <0.0005 | 0.016 |
| Bilirubin_direct_median | 0.016 | <0.0005 | <0.0005 | 1.000 | <0.0005 | 0.003 |
| Bilirubin_direct_IQR | <0.0005 | <0.0005 | <0.0005 | <0.0005 | <0.0005 | 0.025 |
| TotalProtein_median | 1.000 | <0.0005 | <0.0005 | <0.0005 | <0.0005 | 1.000 |
| TotalProtein_IQR | 0.124 | <0.0005 | <0.0005 | <0.0005 | <0.0005 | 1.000 |
| Albumin_median | 0.001 | <0.0005 | <0.0005 | <0.0005 | <0.0005 | 0.159 |
| Albumin_IQR | 0.249 | <0.0005 | <0.0005 | <0.0005 | <0.0005 | 1.000 |
